# Pharmacy inventory management practices and constraints in Asia-Pacific hospitals: a systematic review and qualitative synthesis

**DOI:** 10.1080/20523211.2026.2701922

**Published:** 2026-07-28

**Authors:** Wuan Shuen Yap, Wing Loong Cheong, Li Ling Yeap

**Affiliations:** School of Pharmacy, Monash University Malaysia, Bandar Sunway, Malaysia

**Keywords:** Hospital pharmacy, inventory management, medicine supply, pharmacy operations

## Abstract

**Background:**

Persistent medicine shortages across the Asia-Pacific region suggest variability in hospital inventory management capacity. Despite available guidelines, variations exist across hospitals in adopting recommended inventory management practices. Thus, this study aimed to identify existing inventory management methods, evaluate related challenges, and highlight recommendations for improvement.

**Methods:**

Searches were performed to identify relevant sources from 39 countries on CINAHL, Cochrane, Embase, PubMed, Scopus, Business Source Complete, and Emerald Insight. Non-indexed literature was retrieved through Google Scholar and general Google searches. Backward and forward citation searching was conducted. Data were extracted based on an initial framework comprising three components: planning and procurement, storage and stock management, and staff management. The framework was later expanded through inductive coding to include 12 subcomponents. Findings were narratively synthesised to describe inventory management methods and their impact on medicine supply. Methodological quality of the included empirical publications was assessed using the Mixed Methods Appraisal Tool (MMAT).

**Results:**

Seventy-two publications from 20 countries were included. Practices varied between hospitals even within the same country. Hospitals shared common inventory management challenges that weakened medicine supply systems. A conceptual framework was developed to outline three key themes: systemic constraints, infrastructural limitations, and staff skill gaps. Overcoming systemic constraints requires streamlining expenditure and enhancing central distribution systems. Implementing multi-supplier contracts may be effective at stabilising supply. Infrastructural improvements in storage space and inventory tracking systems may bolster medicine availability. Addressing staff skill gaps requires significant investment in training programmes to strengthen adherence to effective inventory management methods.

**Conclusion:**

This study mapped key determinants of medicine availability in hospitals, providing guidance on priority areas for intervention to policymakers, hospital leadership, and inventory personnel. Securing medicine supplies in hospitals requires practical inventory management guidelines that account for systemic constraints and infrastructural limitations while empowering staff to optimise pharmacy inventory management practices.

## Introduction

The growth in medicine consumption in the Asia-Pacific region is estimated to be amongst the highest in the world (Aitken et al., 2024). Driven by the growing burden of chronic diseases, ageing-associated illnesses, and the demand for newer, costly medicines across the region, overall medicine consumption is expected to expand at over 3% annually through 2028 (Organisation for Economic Co-operation and Development, 2023).

Hospitals in the region cater to a significant portion of seriously ill patients who require uninterrupted access to life-saving medicines. However, the rising demand for medicines may not be sufficiently fulfilled as shortages continue to be reported in hospitals in both low-income nations such as Bangladesh (Sultana et al., 2025) and Sri Lanka (Jeyassuthan et al., 2025), and high-income nations including Australia (The University of Queensland, 2025).

Various countries in the Asia-Pacific region are particularly vulnerable to disruptions in the global medicine supply chain due to limited domestic pharmaceutical manufacturing capacities (Hafner & Popp, 2011; Kraiselburd & Yadav, 2013). As these countries rely heavily on imported medicines (Jakovljevic et al., 2021; Teo et al., 2016), inefficient pharmacy inventory management may exacerbate the impact of unpredictable supply chain issues, putting hospitals at greater risk of frequent, prolonged, and severe medicine shortages. Such shortages have been demonstrated to increase medical staff workload and healthcare spending (Sallam et al., 2024), and result in poorer patient outcomes due to the delayed provision of healthcare services. At the hospital level, inventory management challenges may delay timely treatment, limit access to essential medicines, and increase the need for therapeutic substitutions (Adak, 2024). Suboptimal inventory management practices have also been identified among the reasons estimated to contribute to up to 40% of wasteful health spending (World Health Organisation, 2010), funds which may have been better utilised to secure increased supplies of medicines in demand.

Efficient pharmacy inventory management, which involves a continuous process of coordinating the procurement, storage, and distribution of pharmaceutical products to ensure uninterrupted availability while minimising wastage or excessive spending (Ali, 2011), is instrumental in mitigating the competing risks of overstocking and understocking medicines. Pharmacy inventory management guidelines commonly recommend established methods such as ‘Always Better Control' – ‘Vital, Essential, Non-essential' (ABC-VEN) analyses or ‘First-Expired, First-Out’ (FEFO) distribution, yet the adoption of these methods has been found to be inconsistent between healthcare facilities (Bhusal et al., 2025), due to limitations in infrastructure, financing, and workforce capacity (Bhusal et al., 2025; Kraiselburd & Yadav, 2013). Evidently, the uneven capabilities of facilities in managing pharmacy inventory highlight a critical need for relevant policies that adequately overcome these constraints and support effective compliance to optimal pharmacy inventory management practices.

In this regard, existing literature lacks consolidated evidence to effectively guide policy decisions in streamlining pharmacy inventory management practices. As such, a comparative synthesis of hospital pharmacy inventory management practices at the regional level would benefit both top-level policymakers and local healthcare personnel in identifying operational needs and limitations and enabling the implementation of successful strategies across facilities operating within diverse healthcare systems.

Employing a qualitative synthesis approach, this systematic review aimed to map existing evidence on pharmacy inventory management practices in hospitals in the Asia-Pacific region, identify common barriers that hamper efficient inventory management, and highlight recommendations to support the development of efficient and resilient pharmacy inventory management systems.

## Methods

### Search strategy

In accordance with the Preferred Reporting Items for Systematic Reviews and Meta-Analyses (PRISMA) 2020 protocol (Page et al., 2021), a comprehensive search was conducted between 30 July and 4 August 2025 to identify publications from 39 countries in the Asia-Pacific region. For this review, the Asia-Pacific region was defined using the World Health Organisation classifications for the South-East Asia and Western Pacific regions (World Health Organisation, 2024) (see Supplemental Appendix 1 for the full list of countries). Keywords used included variations of ‘hospital’, ‘pharmacy’, and ‘inventory management’ combined with country names. The full list of concept terms and database-specific search strings is provided in Supplemental Appendix 2. These were adapted to the syntax and controlled vocabulary of each database.

In addition to database searches on CINAHL, Cochrane, Embase, PubMed, Scopus, Business Source Complete, and Emerald Insight, searches were also conducted on Google Scholar and the Google search engine to capture non-indexed literature. No date range or language restrictions were imposed. Following screening, backward and forward citation searching was also performed for all eligible publications. Records retrieved from all sources were collated, and duplicates were identified and removed before screening. The protocol for this review was registered with PROSPERO (CRD420251081261).

### Literature selection

**Figure 1.**
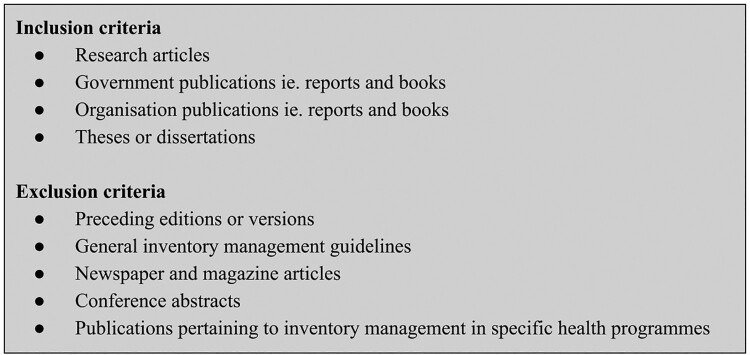
Inclusion and exclusion criteria applied during literature selection.

Peer-reviewed articles, theses and dissertations, and documents issued by relevant organisations and government bodies describing or examining pharmacy inventory management practices in hospitals were included. Only the most recent publication was retained if multiple versions were available. Titles and abstracts were screened first, followed by full-text review. Screening and eligibility assessments were independently completed by two reviewers, with disagreements resolved by discussion. The full inclusion and exclusion criteria are presented in Figure 1.

### Data extraction and analysis

Two reviewers independently extracted data and cross-checked datasets for consistency. Disagreements were resolved through discussion, with a third reviewer consulted when needed. Both reviewers used a pre-defined Microsoft Excel spreadsheet to extract key study characteristics, such as study design, settings, and location.

**Figure 2.**
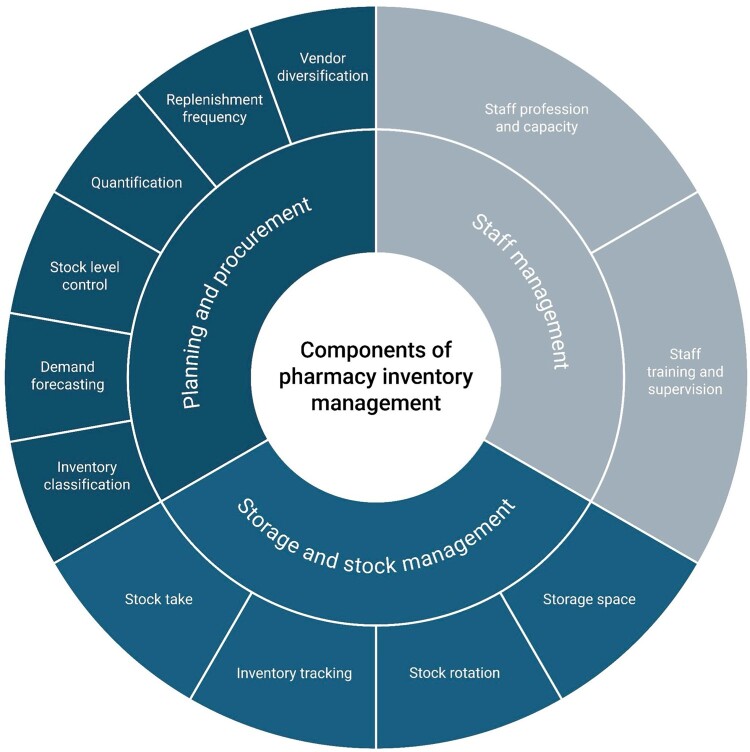
Operational framework of pharmacy inventory management, comprising three core components and 12 subcomponents.

The initial extraction of reported inventory management practices was informed by the works of Sirisawat et al. (2024) and Hlaing and Lat (2024), comprising three main components: (i) planning and procurement, (ii) storage and stock management, and (iii) staff management. Given the breadth of the research objectives, an inductive approach was subsequently undertaken to expand the operational framework with emerging subcomponents, as shown in Figure 2. Where available, the impact of reported inventory management practices on medicine availability was also included in the data extraction matrix. The full data extraction matrix is provided in Supplemental Appendix 4.

### Data synthesis and quality appraisal

Due to the heterogeneity of study designs, methodological quality of the empirical publications was assessed using the Mixed Methods Appraisal Tool (MMAT) (Hong et al., 2018), with methodological quality rated as high (all five criteria met), moderate (at least four criteria met), or low (less than four criteria met). A qualitative thematic synthesis was then undertaken, guided by the approach of Thomas and Harden (2008), involving the coding of extracted data followed by iterative theme development. The country setting and data source type provided context when interpreting and comparing findings across publications. Any discrepancies in the quality appraisal and data synthesis process were resolved by ongoing discussion between the reviewers until a consensus was reached.

### Ethical consideration

Ethical approval was not required, as this systematic review used published literature and involved no primary human data collection.

## Results

**Figure 3.**
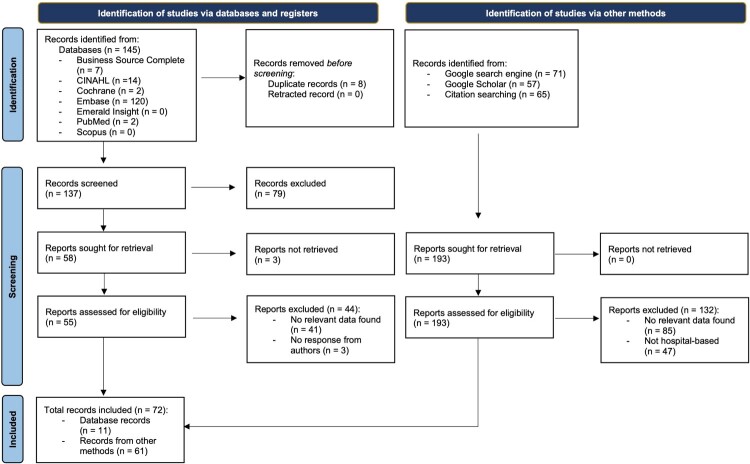
PRISMA 2020 flow diagram of study selection.

Database and grey literature searches identified 273 publications. Citation searching yielded a further 65 records. Most of these publications were read in full, except when title screening was sufficient to determine whether the publication was relevant. In total, 72 publications met the inclusion criteria (Figure 3).

**Figure 4.**
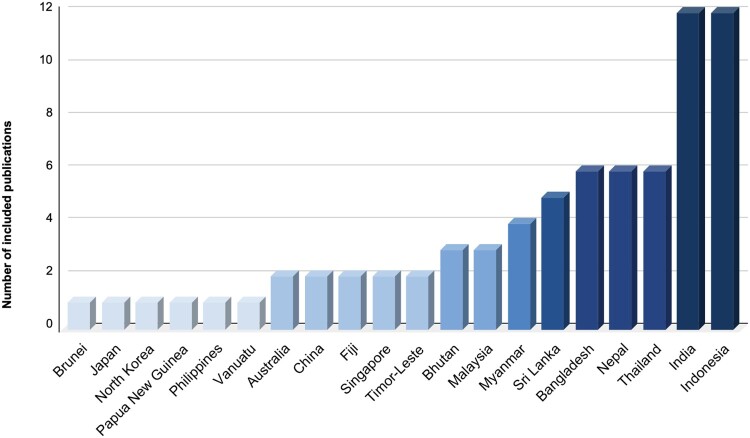
Number of included publications by country.

Relevant publications were identified for 20 of the 39 countries searched. India and Indonesia contributed the highest number of publications (Figure 4). Included publications were published from 2005 to the search date. Study designs were predominantly mixed methods (45.8%), followed by qualitative (30.5%) and quantitative (23.6%) approaches.

The scope of the publications varied, with some describing several subcomponents of inventory management, whereas others addressed only a limited number. Across the 12 subcomponents included in the data extraction sheet, data was available only for several countries per subcomponent. Gathered data provided descriptions of pharmacy inventory management in public and private hospitals. When publications included both hospital and non-hospital institutions in their investigation, care was taken to only include findings gathered in hospital settings. Assessing the included publications with MMAT, 17 publications were rated ‘high’, five were rated ‘moderate’, and the remaining 50 were given a ‘low’ rating (see Supplemental Appendix 3), indicating variable methodological quality across the included evidence. Methodological quality was limited mainly due to incomplete reporting of research procedures and convenience sampling that limited sample sizes and representativeness. The characteristics and methodological quality of the included publications are summarised in Table 1.

**Table 1.** Characteristics and methodological quality of the included publications.

Country	Publication (Reference)	Source type	Site count	Location	Hospital type	Design	Methodological quality (MMAT assessment)
Australia	Shrestha (2016)	Thesis	Multi-centre	4 states and 2 territories in Australia	Public	Qualitative	High
	Ziaee et al. (2023)	Journal article	Multi-centre	N/A	Public	Qualitative	High
Bangladesh	Akhter et al. (2022)	Journal article	Multi-centre	Jashore, Satkhira, Khulna, Kushtia, and Magura	Public and private	Quantitative	Low
	Kochi et al. (2022)	Journal article	Single centre	Dhaka	Public	Quantitative	Low
	Paul et al. (2015)	Journal article	Multi-centre	Bogra, Dhaka, and Rajshahi	Public and private	Qualitative	Low
	Sultana et al. (2025)	Journal article	Multi-centre	Rajshahi	Public	Quantitative	Low
	World Health Organisation (2014a)	Report	Multi-centre	Two divisions in Bangladesh	Public	Mixed methods	Low
	Zahan et al. (2022)	Journal article	Single centre	Dhaka	Public	Mixed methods	Low
Bhutan	Ministry of Health Royal Government of Bhutan (2024)	Report	Multi-centre	N/A	Public	Mixed methods	Low
	Thinley et al. (2017)	Book	N/A*	N/A	Public	Mixed methods	Low
	World Health Organisation (2015a)	Report	Multi-centre	Two regions in Bhutan	Public	Mixed methods	Low
Brunei Darussalam	Ali Hazis et al. (2023)	Journal article	Multi-centre	Belait, Brunei Muara, Temburong, and Tutong	Public	Quantitative	High
China	Wu et al. (2015)	Journal article	Multi-centre	Jiujiang	Public	Mixed methods	Low
	Yang et al. (2016)	Journal article	Multi-centre	Baoji, Xi’an, Xianyang, Shangluo, Weinan, and Yulin	Public	Qualitative	High
Fiji	Roberts et al. (2011)	Book	N/A*	N/A	Public and private	Mixed methods	Low
	Walker et al. (2017)	Journal article	Multi-centre	Suva	Public	Qualitative	Low
India	Chand et al. (2022)	Journal article	Single centre	Karnataka	Private	Mixed methods	Low
	Chokshi et al. (2015)	Journal article	Multi-centre	17 districts in Bihar and 18 districts in Tamil Nadu	Public	Mixed methods	Low
	Dixit et al. (2022)	Journal article	Multi-centre	Rajasthan	Public	Qualitative	Moderate
	Iqbal et al. (2016)	Journal article	Multi-centre	Srinagar	Public	Quantitative	Low
	Jaju et al. (2023)	Journal article	Single centre	N/A	Public	Quantitative	High
	Kant et al. (2015)	Journal article	Single centre	Faridabad	Public	Quantitative	High
	Khembhavi et al (2019)	Journal article	Single- centre	Mumbai	Public	Mixed methods	Low
	Manivannan et al. (2024)	Report	Multi-centre	Tamil Nadu	Public	Qualitative	Low
	Meenu et al. (2015)	Journal article	N/A*	Kerala	Public	Qualitative	Low
	Prinja et al. (2015)	Journal article	Multi-centre	12 districts in the states of Haryana and Punjab	Public	Mixed methods	Low
	Singh et al. (2013)	Journal article	Multi-centre	Kerala, Maharashtra, Odisha, Punjab, and Tamil Nadu	Public	Mixed methods	High
	Vashistha et al. (2018)	Journal article	Multi-centre	Bharatpur	Public	Mixed methods	Low
Indonesia	Anggriani et al. (2020)	Journal article	Multi-centre	Cilegon and Jakarta	Public and private	Quantitative	High
	Asthariq et al. (2022)	Journal article	Single centre	Lhokseumawe	Public	Mixed methods	Low
	Fauzia et al. (2024)	Journal article	Single centre	N/A	Public	Qualitative	Moderate
	Ghozali et al. (2021)	Journal article	Single centre	Magelang	Public	Quantitative	High
	Hakim (2021)	Journal article	Single centre	Dumai	Public	Qualitative	Low
	Hakim & Ulfah (2019)	Journal article	Single centre	South Jakarta	Public	Mixed methods	Low
	Herman et al. (2009)	Journal article	Multi-centre	Bangka, Belitung, Kupang, Ende, Kota Waringin Barat, Jayapura, Palangkaraya, and Gorontalo	Public	Qualitative	Low
	Nasution et al. (2022)	Journal article	Single centre	Lhokseumawe	Public	Mixed methods	Low
	Nelwan et al. (2023)	Journal article	Single centre	Bitung	Public	Qualitative	Low
	Rachmania & Basri (2013)	Journal article	Single centre	N/A	Public	Mixed methods	Low
	Soraya et al. (2022)	Journal article	Single centre	Yogyakarta	Public	Mixed methods	Low
	Sri Rezeki et al. (2022)	Journal article	Single centre	North Sumatera	Public	Qualitative	Low
Japan	Awaya et al. (2005)	Journal article	Single centre	N/A	Public	Quantitative	Moderate
Malaysia	Ahmad Yusri 2025)	Thesis	Single centre	Kedah	Public	Qualitative	Low
	Mahyadin (2018)	Thesis	Multi-centre	Nationwide	Public	Quantitative	Low
	Shah et al. (2023)	Journal article	Multi-centre	Selangor	Public	Quantitative	Moderate
Myanmar	Aye & Anantachoti (2020)	Journal article	Multi-centre	N/A	Public	Mixed methods	Low
	Than et al. (2014)	Book	N/A	N/A	Public	Mixed methods	Low
	Thazin (2020)	Thesis	Single centre	Rakhine	Public	Mixed methods	High
	World Health Organisation (2014b)	Report	Multi-centre	2 regions in Myanmar	Public	Mixed methods	Low
Nepal	Adhikari et al. (2024)	Journal article	Multi-centre	Bagmati	Public	Qualitative	High
	Bhusal et al. (2025)	Journal article	Multi-centre	Bagmati, Lumbini, and Madhesh	Public	Quantitative	Low
	Development Resource Center (2012)	Report	Multi-centre	Dailekh, Kanchanpur, Mahottari, Mustang, and Sankhuwasabha	Public	Mixed methods	High
	Shrestha (2016)	Thesis	Multi-centre	4 major regions in Nepal	Public	Qualitative	High
	Shrestha et al. (2018)	Journal article	Multi-centre	4 regions in Nepal	Public and private	Qualitative	High
	World Health Organisation (2015b)	Report	Multi-centre	2 regions in Nepal	Public and private	Mixed methods	Low
North Korea	Holloway (2012a)	Report	Multi-centre	Pyongyang and neighbouring provinces	Public	Mixed methods	Low
Papua New Guinea	Brown & Gilbert (2014)	Journal article	Multi-centre	3 areas in Papua New Guinea	Public	Qualitative	High
Philippines	Parilla et al. (2022)	Journal article	Multi-centre	Ilocos Norte	Public	Quantitative	Low
Singapore	Kumar et al. (2008)	Journal article	Multi-centre	Singapore (nationwide)	N/A	Mixed methods	Low
	Pan & Pokharel (2007)	Journal article	Multi-centre	Singapore (nationwide)	Public and private	Quantitative	Moderate
Sri Lanka	Bopage et al. (2024)	Journal article	Multi-centre	Monoragala	Public	Quantitative	High
	Jeyassuthan et al. (2025)	Journal article	Multi-centre	Western province of Sri Lanka	Public	Mixed methods	Low
	Ranga (2021)	Thesis	Multi-centre	Western province of Sri Lanka	Public and private	Qualitative	Low
	Sri Lanka Association of Clinical Pharmacology and Therapeutics (2023)	Report	Multi-centre	N/A	Public	Mixed methods	Low
	World Health Organisation (2016a)	Report	Multi-centre	2 regions in Sri Lanka	Public	Mixed methods	Low
Thailand	Chanpuypetch & Kritchanchai (2020)	Journal article	Multi-centre	N/A	Public and private	Qualitative	High
	Kanyakam et al. (2018)	Journal article	Single centre	Mahasarakham	Public	Quantitative	Low
	Kritchanchai & Meesamut (2015)	Journal article	Single centre	N/A	Public	Mixed methods	Low
	Srizongkhram et al. (2021)	Journal article	Single centre	Samutsakorn	Private	Mixed methods	Low
	Theptong (2010)	Thesis	Single centre	Maharakham	Private	Qualitative	Low
	World Health Organisation (2016b)	Report	Multi-centre	2 regions in Thailand	Public	Mixed methods	Low
Timor-Leste	Holloway (2012b)	Report	Multi-centre	3 districts in Timor-Leste	Public	Mixed methods	Low
	Norris et al. (2007)	Journal article	Multi-centre	N/A	Public	Qualitative	Low
Vanuatu	Brown & Gilbert (2012)	Journal article	Multi-centre	3 provinces in Vanuatu	Public	Qualitative	High

N/A: Information not available. N/A*: Not applicable.

### Planning and procurement

#### Inventory classification

Inventory classification methods are systematic approaches to categorising inventory items according to specific criteria such as value, criticality, or consumption rate (May et al., 2017). By grouping the items, tailored inventory control strategies can be applied to optimise budget allocations and stock levels. Among the publications reviewed, only six discussed inventory classification methods in inventory planning [Bangladesh (Kochi et al., 2022; Zahan et al., 2022), India (Chand et al., 2022; Jaju et al., 2023) and Indonesia (Nelwan et al., 2023; Soraya et al., 2022)].

In two neighbouring Bangladeshi public college hospitals, the value-based inventory classification method, ABC analysis, was used to guide procurement decisions (Kochi et al., 2022; Zahan et al., 2022). In India, a combination of ABC and ‘Vital, Essential, Desirable’ (VED) analyses was utilised by a private charitable hospital that complied highly with national quality standards (Chand et al., 2022). ABC-VED analyses were utilised by an Indonesian public hospital to make budget adjustments. Subsequently, the hospital also considered the consumption rate of items, conducting ‘Fast-, Slow-, and Non-moving’ (FSN) analysis to ensure the continuous availability of drugs with high consumption (Nelwan et al., 2023).

Pharmacists in a rural Indian public hospital found the ABC-VED matrix to be an effective inventory planning method (Jaju et al., 2023). In contrast, not applying inventory classification methods during inventory planning was found to be a contributing factor to medicine supply disruptions in a small, Indonesian non-profit hospital (Soraya et al., 2022).

#### Demand forecasting

Demand forecasting involves making predictions of the stock quantity required over a future period to optimise resource planning and reduce stock-outs (Ingle et al., 2021). Forecasting future medicine demand was only described in publications discussing public hospitals in four countries (Table 2). In general, forecasts were completed annually based on past consumption data.

**Table 2.** Demand forecasting methods used in hospital pharmacy settings in Asia-Pacific countries.

Location		Procurement model	Consumption data used	Forecasting frequency	Demand projection vs previous year
Bhutan		Centralised^1^	1 year^1^	Annual^1^	+116%^1^
India	Mumbai	Centralised^2^	1 year^2^	Biennial^2^	+15-20%^2^
	Kerala	Centralised^3^	1 year^3^	Annual^3^	+10-15%^3^
	Maharashtra	Decentralised^3^	1 year^3^	Annual^3^	+10%^3^
	Tamil Nadu	Mixed^3^	1 year^3^	Annual^3^	+10%^3^
	Odisha	Mixed^3^	N/A	Annual^3^	N/A
	Punjab	Decentralised^3^	N/A	Annual^3^	N/A
	Rajasthan	Centralised^4^	N/A	Annual^4^	N/A
Sri Lanka		Centralised^5^	N/A	Annual^5^	+10%^5^, +10-15%^6^
Thailand		Mixed^7^	3 years^8^	Every 3 years, annual plan derived^7^	N/A

Superscript numbers refer to cited references. N/A: Information not available. Data sources: ^1^World Health Organisation (2015a), ^2^Khembhavi et al. (2019), ^3^Singh et al. (2013), ^4^Dixit et al. (2022), ^5^Sri Lanka Association of Clinical Pharmacology and Therapeutics (2023), ^6^World Health Organisation (2016a), ^7^World Health Organisation (2016b), ^8^Kanyakam et al. (2018).

Demand projections typically trended upwards between 10% and 15% above the previous year’s consumption in Indian (Singh et al., 2013) and Sri Lankan hospitals (Sri Lanka Association of Clinical Pharmacology and Therapeutics, 2023; World Health Organisation, 2016a). In comparison, one Mumbai hospital projected a 15–20% increase in the medicine quantity needed (Khembhavi et al., 2019), as forecasts accounted for a two-year period. Due to previous experiences where suppliers delayed deliveries or defaulted on orders, Bhutan’s hospitals were described as inflating quantities required to 26 months’ worth in a one-year-period forecast (World Health Organisation, 2015a), representing a 116% increase. In Thai hospitals, triennial forecasts were formulated based on the average usage calculated from the past three years (Kanyakam et al., 2018; World Health Organisation, 2016b) and were used to develop an annual procurement plan (World Health Organisation, 2016b), seemingly without any upward adjustment as observed in other countries.

#### Quantification

Actual quantities were determined during the order placement process. Quantification decisions appeared to be influenced by three factors: past consumption, budget availability, and seasonal demand. The most common factor was past consumption, which influenced quantification of medicine orders in hospitals in 12 countries [Australia (Ziaee et al., 2023), Bangladesh (Kochi et al., 2022; World Health Organisation, 2014a), China (Wu et al., 2015), Indonesia (Asthariq et al., 2022; Fauzia et al., 2024; Hakim & Ulfah, 2019; Nelwan et al., 2023; Rachmania & Basri, 2013; Soraya et al., 2022), Myanmar (Thazin, 2020; World Health Organisation, 2014b), Nepal (Adhikari et al., 2024; Shrestha et al., 2018), Papua New Guinea (Brown & Gilbert, 2014), Philippines (Parilla et al., 2022), Singapore (Pan & Pokharel, 2007), Sri Lanka (Sri Lanka Association of Clinical Pharmacology and Therapeutics, 2023; World Health Organisation, 2016a), Thailand (Kritchanchai & Meesamut, 2015; Theptong, 2010), Timor-Leste (Holloway, 2012b)]. Budget balances constrained the medicine replenishment in hospitals in Bangladesh (Kochi et al., 2022), India (Iqbal et al., 2016), and Indonesia (Nelwan et al., 2023). One study noted that Nepalese hospitals accounted for seasonal variations by buying more in anticipation of outbreaks or natural disasters as the seasons cycle (Shrestha et al., 2018).

Situational analysis reports found that Timor-Leste hospitals (Holloway, 2012b) and smaller Nepalese hospitals (World Health Organisation, 2015b) generally did not apply standardised methods to calculate the quantities required, leading to inventory imbalances. A similar challenge was described in Papua New Guinea, where despite the availability of quantification guidelines, inventory personnel preferred to make estimates at their own discretion (Brown & Gilbert, 2014). A Nepalese hospital (Adhikari et al., 2024) replenished stocks through arbitrary estimates based on past consumption, similar to a Thai hospital (Theptong, 2010). Furthermore, some hospitals [Indonesia (Hakim & Ulfah, 2019; Rachmania & Basri, 2013; Soraya et al., 2022), Thailand (Kritchanchai & Meesamut, 2015)] were found to purchase more supplies without evidence-based justification, resulting in excess stock.

Concerns about unreliable supply encouraged over-ordering. In one Timorese public hospital, it was observed that hospital staff estimated needs at their discretion and inflated order quantities to receive more supplies from the central warehouse. The deliberate excess ordering was spurred by past experiences when the warehouse sent fewer supplies than expected. Nonetheless, aware of these practices and constrained by limited stock, the warehouse would reciprocate by sending reduced quantities, but at times, the hospitals would end up with excess stock (Norris et al., 2007). Similar observations of stockpiling were reported in a situational analysis report on three districts in Timor-Leste (Holloway, 2012b). Additionally, infrequent episodes of stockpiling were reported in a North Indian hospital as staff felt a need to exhaust the monthly budget they were assigned, indenting excess stock in case of future shortages (Kant et al., 2015).

Furthermore, quantification errors were linked to the omission of stock balances during calculations [Bangladesh (World Health Organisation, 2014a), Indonesia (Fauzia et al., 2024), Papua New Guinea (Brown & Gilbert, 2014), Sri Lanka (Sri Lanka Association of Clinical Pharmacology and Therapeutics, 2023). Adjustments for stock-out periods were overlooked [Myanmar (Thazin, 2020; World Health Organisation, 2014b), Sri Lanka (Sri Lanka Association of Clinical Pharmacology and Therapeutics, 2023; World Health Organisation, 2016a). Although relevant data were available, it was not used to inform quantification exercises [Sri Lanka (Sri Lanka Association of Clinical Pharmacology and Therapeutics, 2023), Vanuatu (Brown & Gilbert, 2012)]. Inaccurate or lack of recordkeeping also hindered effective quantification [Australia (Ziaee et al., 2023), Bangladesh (World Health Organisation, 2014a), Fiji (Walker et al., 2017)], a challenge which may be overcome using electronic inventory systems, as observed in a recent study of Sri Lankan hospitals (Jeyassuthan et al., 2025).

#### Stock level control

To maintain adequate supply, some hospitals in Malaysia (Shah et al., 2023) and Thailand (Kritchanchai & Meesamut, 2015; Srizongkhram et al., 2021) applied the minimum-maximum policy, a method involving the replenishment of stock to the preset maximum level when stock levels fall to the minimum limit (Hernandoko & Widyo Laksono, 2023).

Elsewhere, hospitals only specified either a maximum or minimum stock level. A maximum of 14 days of stock was maintained in Singaporean hospitals (Pan & Pokharel, 2007) compared to 15 days in Rajasthan, India (Vashistha et al., 2018), and 6 months in Nepal (Development Resource Center, 2012). On the other hand, in Bangladesh (Zahan et al., 2022) and Karnataka, India (Chand et al., 2022), only minimum stock levels were predetermined.

Kritchanchai and Meesamut observed that one hospital placed orders when the stock balance was at 70-80% of the estimated total monthly usage (2015). Actual usage patterns were not considered by the inventory staff, resulting in over-and-understocking (Kritchanchai & Meesamut, 2015; Srizongkhram et al., 2021).

#### Replenishment frequency

**Figure 5.**
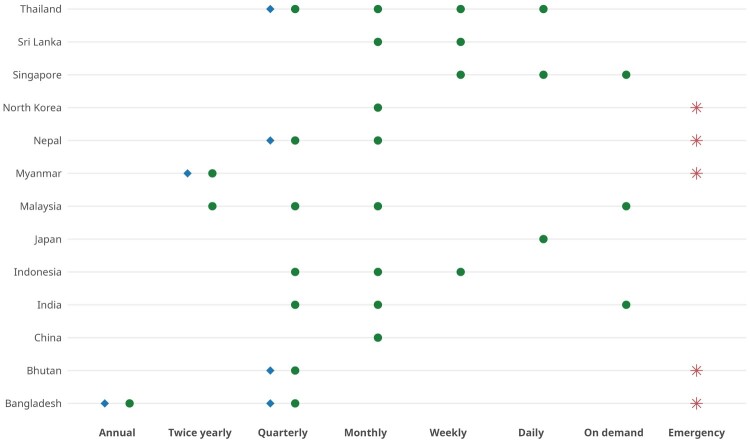
Reported hospital ordering frequencies, including emergency ordering, across Asia-Pacific countries. Blue diamonds indicate central requisition schedules, which refer to ordering through a central supply or warehouse system; green circles indicate routine hospital ordering patterns, which refer to regular ordering by hospitals; red stars indicate emergency or ad-hoc ordering, which refers to urgent or unscheduled orders placed outside routine ordering schedules. Emergency order frequency (where reported): Bangladesh 2–3 times per quarter; Bhutan >4 times per year; Myanmar 2–3 times weekly to 2–3 times yearly; Nepal unspecified; North Korea 1–2 times per quarter]. [Data sources: Bangladesh (Kochi et al., 2022; World Health Organisation, 2014a), Bhutan (Thinley et al., 2017; World Health Organisation, 2015a), China (Wu et al., 2015), India (Dixit et al., 2022; Iqbal et al., 2016; Jaju et al., 2023), Indonesia (Nasution et al., 2022; Rachmania & Basri, 2013; Soraya et al., 2022), Malaysia (Ahmad Yusri, 2025; Shah et al., 2023), Myanmar (Than et al., 2014; Thazin, 2020; World Health Organisation, 2014b), Nepal (Adhikari et al., 2024; Bhusal et al., 2025; World Health Organisation, 2015b), North Korea (Holloway, 2012a), Singapore (Pan & Pokharel, 2007), Sri Lanka (World Health Organisation, 2016a), Thailand (Srizongkhram et al., 2021; World Health Organisation, 2016b)].

Replenishment frequencies varied widely across countries and were shaped by supply chain structures (Figure 5). Burmese public hospitals followed a requisition schedule, receiving supplies from government warehouses. Insufficient twice-yearly requisitions resulted in emergency orders as frequent as thrice weekly or between two to three times yearly (World Health Organisation, 2014b). Additionally, long intervals between routine orders were cited to cause stock-outs in one hospital (Thazin, 2020). In Bangladesh, emergency orders were made particularly when delivery delays occurred (World Health Organisation, 2014a).

Smaller hospitals in Sri Lanka generally ordered monthly, whereas larger hospitals with higher consumption placed weekly orders (World Health Organisation, 2016a). Thai hospitals also reported daily and weekly ordering to meet high demand, despite recommendations to order every three months (World Health Organisation, 2016b). Daily ordering was also practised in a Japanese hospital using an automated inventory system that effectively reduced workload (Awaya et al., 2005).

#### Vendor diversification

The number of contracted vendors differed amongst hospitals. Supply contracts were awarded to a sole vendor in some hospitals in Australia (Ziaee et al., 2023) and Indonesia (Anggriani et al., 2020). Indonesian public hospitals were reported to prioritise purchases from assigned vendors listed on a government procurement portal, although vendors who failed to meet contractual obligations faced no penalties (Anggriani et al., 2020).

By contrast, the Indian states of Tamil Nadu and Kerala awarded contracts to multiple suppliers as a strategy to address potential shortages and ensure continuous supply (Chokshi et al., 2015; Manivannan et al., 2024; Meenu et al., 2015). In Tamil Nadu, suppliers who did not win the tender were recruited as reserve suppliers (Manivannan et al., 2024). In Kerala, when several vendors quoted prices close to the lowest bid, contracts were shared (Meenu et al., 2015). Studies from Singapore found that hospitals typically had two to three vendors per item (Pan & Pokharel, 2007), and in some cases, up to six (Kumar et al., 2008).

### Storage and stock management

#### Storage space

Designated drug storage areas were found to be of adequate size in some hospitals [Bangladesh (Kochi et al., 2022), India (Vashistha et al., 2018), Indonesia (Nelwan et al., 2023)]. Storage space was considered to be lacking when adequate stock levels could not be maintained in the hospitals, as seen in Bhutan (World Health Organisation, 2015a), India (Chand et al., 2022; Prinja et al., 2015), Indonesia (Herman et al., 2009), Nepal (Adhikari et al., 2024; Bhusal et al., 2025), Sri Lanka (World Health Organisation, 2016a), and Timor-Leste (Norris et al., 2007). Space constraints led to medicines inappropriately stored directly on the floor (Prinja et al., 2015) and impeded effective FEFO practices (Adhikari et al., 2024).

#### Stock rotation

Stock rotation methods govern how medicines are withdrawn from storage for use or consumption (Alamsyah et al., 2025; Hertog et al., 2014). The FEFO method, which emphasises the withdrawal of products with the shortest shelf-life (Alamsyah et al., 2025), was the most common stock rotation method implemented in hospital pharmacies [Bangladesh (Kochi et al., 2022; Sultana et al., 2025), Brunei Darussalam (Ali Hazis et al., 2023), India (Prinja et al., 2015), Indonesia (Hakim, 2021; Nelwan et al., 2023; Sri Rezeki et al., 2022), Myanmar (Thazin, 2020), Papua New Guinea (Brown & Gilbert, 2014), Sri Lanka (Bopage et al., 2024), Thailand (Kanyakam et al., 2018; Theptong, 2010; World Health Organisation, 2016b)].

Alternatively, some hospitals applied the FIFO method, which prioritises the withdrawal of stock items according to when items arrived in storage (Alamsyah et al., 2025). Hospitals either applied the FIFO method on its own [India (Jaju et al., 2023; Vashistha et al., 2018), Indonesia (Herman et al., 2009)] or in combination with the FEFO method [Bangladesh (Kochi et al., 2022), Indonesia (Nelwan et al., 2023; Sri Rezeki et al., 2022), Thailand (World Health Organisation, 2016b)]. Two studies observed that pharmacy staff reported good compliance with stock rotation policies [Bangladesh (Sultana et al., 2025), India (Prinja et al., 2015)].

The FEFO method was used as a measure to prevent medicine wastage at a Bruneian hospital (Ali Hazis et al., 2023) whereas pharmacists at an Indian tertiary hospital considered the FIFO policy to be effective at preventing expiry (Jaju et al., 2023). A Nepalese study observed that expired medicines accumulated in hospitals that did not practise any stock rotation policy (Bhusal et al., 2025).

#### Inventory tracking

Compared to digital or hybrid systems, manual inventory systems were more commonly used with stock movement recorded using bin cards and stock ledgers (Table 3). Manual recordkeeping was of satisfactory quality in Indonesia (Ghozali et al., 2021; Hakim, 2021; Herman et al., 2009; Nasution et al., 2022) and Myanmar (World Health Organisation, 2014b). In Bangladesh, pharmacy staff in a single-centre study regarded their recordkeeping practices as competent (Kochi et al., 2022), but a broader World Health Organisation assessment identified errors in manual records (World Health Organisation, 2014a). When documenting inventory, pharmacy staff in a Thai hospital omitted important details such as batch expiry dates (Chanpuypetch & Kritchanchai, 2020). Inventory personnel in Papua New Guinea neglected updating stock cards and following standard recordkeeping procedures (Brown & Gilbert, 2014).

**Table 3.** Types of inventory systems utilised in hospital pharmacies.

Country	Manual inventory system (Reference)	Hybrid inventory system (Reference)	Digital inventory system (Reference)
Bangladesh	✓ (Kochi et al., 2022; Sultana et al., 2025; World Health Organisation, 2014a; Zahan et al., 2022)		
Fiji	✓ (Roberts et al., 2011)		✓*(Roberts et al., 2011)
India			✓ (Chand et al., 2022; Khembhavi et al., 2019; Vashistha et al., 2018)
Indonesia	✓ (Ghozali et al., 2021; Hakim & Ulfah, 2019; Hakim, 2021; Herman et al., 2009; Nasution et al., 2022)	✓ (Sri Rezeki et al., 2022)	
Japan			✓ (Awaya et al., 2005)
Malaysia		✓ (Ahmad Yusri, 2025)	
Myanmar	✓ (Thazin, 2020; World Health Organisation, 2014b)		
Nepal		✓ (Adhikari et al., 2024)	✓ (Bhusal et al., 2025)
North Korea	✓ (Holloway, 2012a)		
Papua New Guinea	✓ (Brown & Gilbert, 2014)		
Philippines	✓ (Parilla et al., 2022)		
Singapore			✓ (Pan & Pokharel, 2007)
Sri Lanka	✓ †(World Health Organisation, 2016a)]		✓ (Bopage et al., 2024; Jeyassuthan et al., 2025; World Health Organisation, 2016a)
Thailand	✓ (Chanpuypetch & Kritchanchai, 2020; Theptong, 2010)		✓(Kritchanchai & Meesamut, 2015; World Health Organisation, 2016b)
Timor-Leste	✓ (Holloway, 2012b)		

*Reported in divisional hospitals. † Reported in smaller hospitals.

Electronic inventory systems produced more reliable inventory records in Sri Lankan hospitals (Bopage et al., 2024; Jeyassuthan et al., 2025). Larger hospitals appear to benefit from digitalising their inventory system, reporting improvement in inventory management after implementation (Jeyassuthan et al., 2025). In a Malaysian hospital, digitalisation was also perceived to facilitate inventory management, although electronic records were supplemented with physical bin card records (Ahmad Yusri, 2025).

Indian pharmacists considered introducing barcode technology into medicine inventories beneficial (Jaju et al., 2023). However, Vashistha and colleagues (2018) reported that despite the availability of electronic inventory software, physical reports were retained because of unstable Internet and server connections, illustrating that the effective use of digital systems required robust infrastructure.

#### Stock take

Physical counts were reported to be carried out daily [India (Vashistha et al., 2018), Japan (Awaya et al., 2005), Papua New Guinea (Brown & Gilbert, 2014), Thailand (Theptong, 2010)], monthly [Bangladesh (Sultana et al., 2025), India (Chand et al., 2022), Indonesia (Nelwan et al., 2023), Malaysia (Shah et al., 2023), Myanmar (Thazin, 2020)], or annually [Bangladesh (Kochi et al., 2022)]. Shah et al. reported that although monthly checks were expected, facilities that did not possess adequate manpower to adhere to a monthly schedule instead conducted stock counts every 3 or 6 months (2023). A Thai hospital selected items randomly for daily checks, only undertaking a full count annually (Theptong, 2010). By contrast, a survey of hospitals in Nepal found that stock takes were not regularly conducted, which compromised the accuracy of stock replenishment (Bhusal et al., 2025).

### Staff management

#### Staff profession and capacity

Across 13 countries, while mainly assigned to pharmacists, staff members of different professions were also assigned to inventory management tasks (Table 4).

**Table 4.** Staff professions assigned to inventory tasks in hospital pharmacies.

**Staff profession**	**Country**
Pharmacists	● Australia (Shrestha, 2016) ● Bangladesh (Paul et al., 2015) ● Bhutan (Ministry of Health Royal Government of Bhutan, 2024; World Health Organisation, 2015a) ● India (Kerala) (Singh et al., 2013) ● Indonesia (Asthariq et al., 2022; Nasution et al., 2022) ● Japan (Awaya et al., 2005) ● Malaysia (Ahmad Yusri, 2025; Mahyadin, 2018; Shah et al., 2023) ● Myanmar (Aye & Anantachoti, 2020; World Health Organisation, 2014b) ● Nepal (Shrestha, 2016; Shrestha et al., 2018) ● North Korea (Holloway, 2012a) ● Papua New Guinea (Brown & Gilbert, 2014) ● Sri Lanka (Jeyassuthan et al., 2025) ● Thailand (Theptong, 2010; World Health Organisation, 2016b)
Pharmacy assistants	● Bangladesh (Paul et al., 2015) ● Bhutan (Ministry of Health Royal Government of Bhutan, 2024; World Health Organisation, 2015a) ● Malaysia (Shah et al., 2023) ● Sri Lanka (Jeyassuthan et al., 2025)
Physicians	Sri Lanka (World Health Organisation, 2016a)
Nurses	● Bangladesh (Sultana et al., 2025) ● Papua New Guinea (Brown & Gilbert, 2014)
Storekeepers	● Bangladesh (World Health Organisation, 2014a) ● Indonesia (Nelwan et al., 2023) ● Sri Lanka (World Health Organisation, 2016a)
Administrative staff	● Bangladesh (World Health Organisation, 2014a) ● India (Odisha) (Singh et al., 2013) ● Malaysia (Mahyadin, 2018)

In Bangladesh, graduate pharmacists were observed to be mainly employed in private hospitals (Paul et al., 2015). In contrast, a World Health Organisation report found that public hospitals in two divisions in the country allocated inventory duties to storekeepers. While procurement was tasked to the hospital’s senior management, quantification was reported to be of poor quality (World Health Organisation, 2014a). Pharmacists were also not involved in procurement in hospitals in Jammu and Kashmir (Iqbal et al., 2016), whereas in Odisha, clerks performed procurement tasks in lieu of trained inventory staff (Singh et al., 2013)**.**

One multi-hospital study in the Philippines regarded inventory management skills amongst pharmacy staff to be competent (Parilla et al., 2022). However, other studies observed a need for staff upskilling in various areas of inventory management. A survey by the Ministry of Health of Bhutan revealed that pharmacists demonstrated low mastery of forecasting, procurement, and stock management methods (2024), whereas poor awareness of FIFO/FEFO methods was reported in an Indonesian public hospital (Sri Rezeki et al., 2022). In Fiji, Walker and colleagues (2017) attributed medicine supply disruptions to a shortage of employees trained in medicine purchasing. In hospitals in the Shaanxi province in China, procurement staff were reported to lack practical experience (Yang et al., 2016).

Personnel shortages were also reported to hinder completion of inventory management tasks in Bangladesh (Akhter et al., 2022), Nepal (Shrestha et al., 2018), and Vanuatu (Brown & Gilbert, 2012). Staffing challenges were compounded by the frequent transfer of trained personnel (Adhikari et al., 2024) and difficulties in staff retention (Brown & Gilbert, 2012).

#### Staff training and supervision

Hospitals in three countries reported training pharmacy staff in inventory management [India (Vashistha et al., 2018), Indonesia (Nasution et al., 2022), Malaysia (Shah et al., 2023)]. In contrast, formal training in inventory management was not provided in hospitals in Bangladesh (Kochi et al., 2022; Sultana et al., 2025; Zahan et al., 2022), Indonesia (Sri Rezeki et al., 2022), Nepal (Adhikari et al., 2024; World Health Organisation, 2015b), Papua New Guinea (Brown & Gilbert, 2014), and Sri Lanka (Jeyassuthan et al., 2025; Ranga, 2021). One Sri Lankan study found that about half of the staff possessed only average knowledge due to inadequate training (Jeyassuthan et al., 2025). In Nepal, although inventory training manuals were available, staff did not use them effectively (Bhusal et al., 2025).

Formal training and the usage of inventory management guidelines were associated with perceived competency among pharmacy staff in an Indian hospital (Vashistha et al., 2018). However, competency did not appear to consistently translate into good practices. Despite demonstrating good working knowledge of guidelines, inventory personnel in Papua New Guinea (Brown & Gilbert, 2014) and Vanuatu (Brown & Gilbert, 2012) were found to be reluctant to apply the guidelines in practice. Studies in Papua New Guinea (Brown & Gilbert, 2014) and Sri Lanka (Jeyassuthan et al., 2025) have also identified the lack of supervision as a potential contributor to reduced staff performance and accountability.

## Discussion

**Figure 6.**
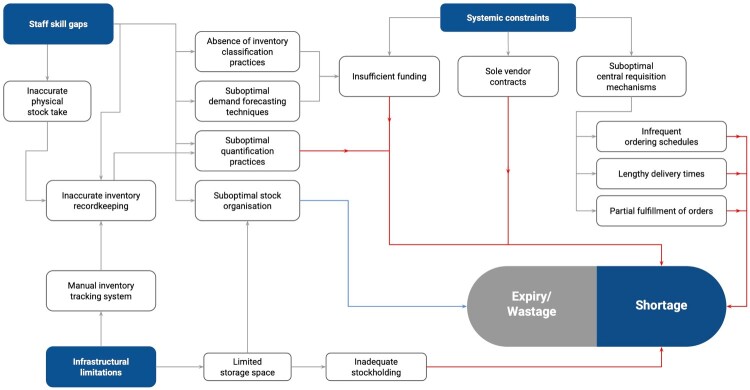
Drivers of inventory imbalances in hospital pharmacies. Staff skill gaps refer to limitations in staff knowledge, competency, or adherence to inventory management practices. Systemic constraints refer to wider procurement, financing, and supply system factors that affect inventory management. Infrastructural limitations refer to physical or technological constraints, including storage capacity and inventory tracking systems. Grey arrows indicate general associations between factors within the inventory management system inferred from the qualitative synthesis. Red arrows represent hypothesised pathways that may contribute to stock shortages. Blue arrows represent hypothesised pathways that may contribute to stock expiries and wastage.

The included literature provided data for only a subset of countries for each of the 12 inventory management subcomponents explored in this review. Nonetheless, this review offers synthesised evidence from hospitals across the Asia-Pacific region and observed variations in practice across and within countries. This review identified three interrelated categories of inventory management challenges associated with stock level imbalances: (i) staff skill gaps, (ii) systemic constraints, and (iii) infrastructural limitations (Figure 6).

Staff skill gaps appeared evident across multiple subcomponents, including inventory classification, demand forecasting, order quantification, stock organisation, stock-taking, and recordkeeping. Although pharmacists were commonly assigned to inventory management, knowledge and competency appeared insufficient in several settings. Although FEFO is more appropriate for perishable products like medicines compared to FIFO, which is better suited for items with longer shelf lives (Alamsyah et al., 2025), several hospitals have reported applying the FIFO method in organising medicine supplies. This suboptimal practice has been observed in both hospitals that relied on manual inventory tracking systems [Bangladesh (Kochi et al., 2022), Indonesia (Herman et al., 2009)] and digital inventory tracking systems [India (Vashistha et al., 2018), Thailand (World Health Organisation, 2016b)]. Moreover, recordkeeping practices in the hospitals that utilised manual inventory tracking systems were considered to be of satisfactory quality [Bangladesh (Kochi et al., 2022), Indonesia (Herman et al., 2009)]. Furthermore, inventory staff perceived FIFO to be effective in preventing medicine expiry [Brunei (Ali Hazis et al., 2023), India (Jaju et al., 2023)]. This indicated that unsuitable stock rotation practices may not be attributable to the type of inventory tracking system utilised or recordkeeping compliance but may partly reflect staff knowledge gaps that warrant appropriate training and education.

Similar to the publications included in this review, a study in Ethiopian public healthcare facilities found that pharmacists lacked awareness of key purposes of inventory management, indicating suboptimal practices (Jobira et al., 2022). The skill gap may be overcome by the provision of upskilling opportunities to bolster staff competency and performance (Gutesa et al., 2024). Moreover, continuous professional development programmes have been shown to improve job satisfaction and staff retention (Barakat & Sallam, 2025). To be effective, these training sessions should be led by engaging trainers who allow adequate participation time for attendees to practise new skills (Jobira et al., 2022). Additionally, staff knowledge levels and the language used should also be accounted for in the design of these training sessions (Walker et al., 2017). Other than training programmes, consistent, supportive senior guidance and supervision have also been shown to improve staff performance (Gutesa et al., 2024; Trap et al., 2001).

Systemic constraints included insufficient medicine budget allocations, the designation of sole suppliers, and suboptimal central requisition systems. These challenges were found to contribute to medicine shortages. Previous research in the European region has highlighted the cost savings generated through central procurement, as bulk purchasing of medicines provided greater negotiating power with vendors (Baldi & Vannoni, 2015; Vogler et al., 2022). However, although central procurement may enable healthcare facilities to purchase more supplies on the same budget, the manner in which distribution was conducted may affect the timely provision of medicines to the facilities. In the countries identified to implement a central medicine procurement and supply system, hospitals were expected to adhere to predetermined requisition schedules where the duration between orders tended to be months-long, suggesting that it may be difficult to determine accurate estimates for longer periods. As a result, hospitals were likely to place several emergency orders before the next procurement cycle, effectively rendering the requisition schedule redundant.

Moreover, delivery times appeared longer for hospitals that received supplies from central warehouses compared to hospitals that purchased medicines on their own, suggesting that central distribution may be subject to administrative delays (Walters, 2025) or hampered by insufficient logistical resources, which limited the system’s ability to service facilities equally regardless of geographical location (Yang et al., 2017). In part, the extended delivery time may have contributed to emergency orders being made while waiting for the initial orders to arrive. Apart from inconsistent delivery times, shortages may also be aggravated by the unequal distribution of supplies (Ranga, 2021; World Health Organisation, 2014a). Notably, these supply uncertainties in central distribution systems may have increased the tendency of inventory staff to accumulate excess stock during the normal requisition process (Holloway, 2012b; Kant et al., 2015; Norris et al., 2007). The evidence indicates a need to streamline and expand the capacity of central requisition systems to ensure supplies are delivered to facilities on time, and at the right quantity.

Shortages have been attributed to the inability of contracted vendors to provide supplies within the expected delivery period, an issue further exacerbated when only a single vendor was appointed. As exemplified by states in India and Singapore, contracting multiple vendors was reported as a strategy to prevent shortages in times when a main vendor was unable to provide adequate supply. This strategy has also been recommended by the Medicines for Europe organisation as part of broader efforts to enhance medicine availability (2023). Additionally, securing multiple suppliers may result in financial savings through the provision of split contracts (Development Resource Center, 2012; Stoermer et al., 2009).

While systemic constraints may have resulted in limited medicine budgets, staff skill gaps in inventory classification and demand forecasting may have contributed to an inefficient utilisation of funds. Although recommended in pharmacy inventory management guidelines from several health ministries in the region (Ministry of Health Malaysia, 2020; Pamela et al., 2019; State Institute of Health & Family Welfare Rajasthan, 2010), it was unclear whether inventory classification guided the planning process in hospital pharmacies, raising the possibility that inventory budgets may not have been apportioned effectively. Furthermore, irrespective of the facility’s procurement model, demand projections were completed yearly and typically inflated between 10% and 20% from the past year’s recorded usage volume. However, the publications reviewed did not address why increased demand was quantified as such, nor were the forecasting methods applied sufficiently described. While adjusting procured quantities during stock replenishment may mitigate forecasting inaccuracies (Zhou et al., 2023), conforming to a ‘historical’ projection estimate may result in insufficient funding or supply provision in the future, particularly in hospitals that rely on a central requisition system with no autonomy or funds to initiate medicine purchases on their own. To produce accurate forecasts, maintaining accurate inventory data is essential (Bilal et al., 2024). Additionally, consideration should be given to factors such as funding availability, current stock levels, and the expected stock levels to be maintained by the end of the planning cycle (World Health Organisation, 2026).

Infrastructural limitations in storage space contributed to inventory imbalances by impeding optimal stockholding (Ghassami & Ghandehary, 2014). Combined with inadequate staff awareness of proper stock rotation methods, the lack of space led to stock expiries. If larger storage spaces cannot be allocated, overcoming space constraints may require improved storage solutions to maximise storage capacity. Alternatively, increased ordering of smaller quantities may be necessary, an option that may only be feasible provided delivery times are reliable, and the availability of supplies guaranteed.

While digital systems were considered to improve the efficiency of inventory management in some hospitals, findings from Indonesian and Burmese hospitals have indicated that manual recordkeeping methods can produce reliable inventory records with consistent recordkeeping practices. When infrastructural limitations permitted only the use of manual systems, staff competency was essential to produce accurate inventory recordkeeping to inform order quantification during stock replenishment. Quantification of future purchases was mainly informed by past consumption data, highlighting the need for regular stock checks to bolster the accuracy of stock records (Tundura & Wanyoike, 2016). The use of emerging digital tools such as barcode-enabled tracking and automated inventory monitoring may improve stock visibility and support timely procurement decisions (Abimanyu et al., 2025). Upgrading to an electronic inventory system may enable leveraging real-time data to determine purchase quantities at greater accuracy (Kochi et al., 2022). However, such measures may only be viable in more affluent healthcare systems supported by robust digital infrastructure and investment in effective staff training (Abimanyu et al., 2025).

Quality improvement measures such as the adoption of digital systems, appropriate stock rotation methods, and waste reduction strategies have been shown to enhance the operational performance of hospital pharmacies (Sallam, 2024). Yet, these initiatives to improve inventory management outcomes remain contingent on addressing underlying systemic and infrastructural constraints. Enhancing inventory management practices may also require significant investment in capacity-building programmes amongst pharmacy staff to ensure the sustained implementation of efficient and effective pharmacy inventory management practices.

To our knowledge, this is the first review synthesising hospital pharmacy inventory management practices across the Asia-Pacific region. Strengths of this review include its multinational scope and inclusion of non-indexed literature. However, evidence gaps remain. Low ratings in the MMAT assessment reflect limitations in the available evidence base, suggesting the need for more robust study designs in this area. Few studies linked inventory practices to clinical outcomes, patient costs, or health system performance. As such, future studies to measure the clinical, fiscal, operational, and organisational outcomes of reforming pharmacy inventory management practices may be beneficial in supporting evidence-based decision-making in hospital medicine supply chains.

This review has limitations. Internal hospital documents may have been excluded from our searches. Although formal inter-rater reliability was not calculated, screening and eligibility assessments were undertaken independently by two reviewers, and disagreements were resolved through consensus. As the included sources were published over a 20-year period, the findings may reflect historical and institution-specific conditions. Additionally, the sources predominantly discussed public hospitals, offering limited insight into private hospitals. Thus, this review may not be interpreted as a static representation of current inventory management practices in the region, but rather as indicative of broad patterns reported across diverse contexts. As such, while findings of this review may not be generalised to all countries in the region, the identified challenges provide actionable insights for strengthening pharmacy inventory management across diverse health systems. Beyond describing existing practices, the conceptual framework developed in this review offers a structured approach to support policymakers, hospital personnel, and researchers to assess inventory systems holistically and identify intervention priorities relevant to their settings.

## Conclusion

Pharmacy inventory management in Asia-Pacific hospitals is influenced by interrelated staff skill gaps, systemic constraints, and infrastructural limitations that affect medicine availability. Interventions targeting single inventory components risk shifting inefficiencies elsewhere if system-wide interactions were not considered. Hence, future efforts such as inventory system reforms or tailored upskilling programmes must be practical and implementable. Only by addressing the gaps between formal policy and day-to-day practice can pharmacy inventory staff be empowered to optimise practices and reduce reliance on compensatory workarounds. Recommended solutions should also consider infrastructure and workforce limitations to support adaptive inventory management that safeguard medicine availability and maximise medical resource utilisation across hospitals.

## Supplementary Material

Appendix 4 Data extraction matrix.xlsx

Appendix 2 Search string.docx

Appendix 1 List of countries.docx

Appendix 3 MMAT appraisal.xlsx
