# Supplementary material for: Pharmacy inventory management practices and constraints in Asia-Pacific hospitals: a systematic review and qualitative synthesis
Source: J Pharm Policy Pract. 2026 Jul 28;19(1):2701922. doi: 10.1080/20523211.2026.2701922 (PMC13417656; doi:10.1080/20523211.2026.2701922)
Supplement: Appendix 2 Search string.docx [file JPPP_A_2701922_SM7075.docx]

# Appendix 2. Search string utilised on each database

| **Database** | **Search terms** |
| --- | --- |
| CINAHL Complete | hospital OR “healthcare facility”  AND medicines OR medications OR drugs OR pharmacies OR pharmaceuticals OR (MH "Medicine+") OR (MH "Drugs+")    AND “inventory management” OR “managing inventory” OR “inventory control” OR “controlling inventory” OR “stock management” OR “managing stock” OR “stock control” OR “controlling stock” OR “supply management” OR “managing supply”  AND Bangladesh OR Bhutan OR Democratic People’s Republic of Korea OR North Korea OR India OR Indonesia OR Maldives OR Myanmar OR Nepal OR Sri Lanka OR Thailand OR Timor-Leste OR East Timor OR Australia OR Brunei Darussalam OR Cambodia OR China OR Cook Islands OR Fiji OR Japan OR Kiribati OR Lao People's Democratic Republic OR Laos OR Malaysia OR Marshall Islands OR Micronesia OR Mongolia OR Nauru OR New Zealand OR Niue OR Palau OR Papua New Guinea OR Philippines OR Republic of Korea OR South Korea OR Samoa OR Singapore OR Solomon Islands OR Tokelau OR Tonga OR Tuvalu OR Vanuatu OR Vietnam |
| Cochrane | hospital OR “healthcare facility”  AND medicine OR medication OR drug OR pharmacy OR pharmaceutical    AND “inventory management” OR “managing inventory” OR “inventory control” OR “controlling inventory” OR “stock management” OR “managing stock” OR “stock control” OR “controlling stock” OR “supply management” OR “managing supply”  AND Bangladesh OR Bhutan OR Democratic People’s Republic of Korea OR North Korea OR India OR Indonesia OR Maldives OR Myanmar OR Nepal OR Sri Lanka OR Thailand OR Timor-Leste OR East Timor OR Australia OR Brunei Darussalam OR Cambodia OR China OR Cook Islands OR Fiji OR Japan OR Kiribati OR Lao People's Democratic Republic OR Laos OR Malaysia OR Marshall Islands OR Micronesia OR Mongolia OR Nauru OR New Zealand OR Niue OR Palau OR Papua New Guinea OR Philippines OR Republic of Korea OR South Korea OR Samoa OR Singapore OR Solomon Islands OR Tokelau OR Tonga OR Tuvalu OR Vanuatu OR Vietnam |
| Embase via Ovid  (Embase Classic) | hospital.mp. OR health care facility.mp. OR exp hospital/ OR exp health care facility/  AND medicine.mp. OR medication.mp. OR drug.mp. OR pharmacy.mp. OR pharmaceutical.mp. OR exp medicine/ OR exp drug/    AND inventory management.mp. OR managing inventory.mp. OR inventory control.mp. OR controlling inventory.mp. OR stock management.mp. OR managing stock.mp. OR stock control.mp. OR controlling stock.mp. OR supply management.mp. OR managing supply.mp. OR exp inventory control/  AND Bangladesh OR Bhutan OR Democratic People’s Republic of Korea OR North Korea OR India OR Indonesia OR Maldives OR Myanmar OR Nepal OR Sri Lanka OR Thailand OR Timor-Leste OR East Timor OR Australia OR Brunei Darussalam OR Cambodia OR China OR Cook Islands OR Fiji OR Japan OR Kiribati OR Lao People's Democratic Republic OR Laos OR Malaysia OR Marshall Islands OR Micronesia OR Mongolia OR Nauru OR New Zealand OR Niue OR Palau OR Papua New Guinea OR Philippines OR Republic of Korea OR South Korea OR Samoa OR Singapore OR Solomon Islands OR Tokelau OR Tonga OR Tuvalu OR Vanuatu OR Vietnam |
| PubMed | “hospitals”[mh] OR “healthcare facilities”[mh]  AND “medicine” OR “medication” OR “drug” OR “pharmacy” OR “pharmaceutical” OR "pharmaceutical preparations"[mh]    AND “inventory management” OR “managing inventory” OR “inventory control” OR “controlling inventory” OR “stock management” OR “managing stock” OR “stock control” OR “controlling stock” OR “supply management” OR “managing supply”  AND Bangladesh OR Bhutan OR Democratic People’s Republic of Korea OR North Korea OR India OR Indonesia OR Maldives OR Myanmar OR Nepal OR Sri Lanka OR Thailand OR Timor-Leste OR East Timor OR Australia OR Brunei Darussalam OR Cambodia OR China OR Cook Islands OR Fiji OR Japan OR Kiribati OR Lao People's Democratic Republic OR Laos OR Malaysia OR Marshall Islands OR Micronesia OR Mongolia OR Nauru OR New Zealand OR Niue OR Palau OR Papua New Guinea OR Philippines OR Republic of Korea OR South Korea OR Samoa OR Singapore OR Solomon Islands OR Tokelau OR Tonga OR Tuvalu OR Vanuatu OR Vietnam |
| Scopus | hospital OR “healthcare facility”  AND medicine OR medication OR drug OR pharmacy OR pharmaceutical    AND “inventory management” OR “managing inventory” OR “inventory control” OR “controlling inventory” OR “stock management” OR “managing stock” OR “stock control” OR “controlling stock” OR “supply management” OR “managing supply”  AND Bangladesh OR Bhutan OR Democratic People’s Republic of Korea OR North Korea OR India OR Indonesia OR Maldives OR Myanmar OR Nepal OR Sri Lanka OR Thailand OR Timor-Leste OR East Timor OR Australia OR Brunei Darussalam OR Cambodia OR China OR Cook Islands OR Fiji OR Japan OR Kiribati OR Lao People's Democratic Republic OR Laos OR Malaysia OR Marshall Islands OR Micronesia OR Mongolia OR Nauru OR New Zealand OR Niue OR Palau OR Papua New Guinea OR Philippines OR Republic of Korea OR South Korea OR Samoa OR Singapore OR Solomon Islands OR Tokelau OR Tonga OR Tuvalu OR Vanuatu OR Vietnam |
| Business Source Complete (via EBSCOhost) | hospital OR “healthcare facility”  AND medicines OR medications OR drugs OR pharmacies OR pharmaceuticals OR (MH "Medicine+") OR (MH "Drugs+")    AND “inventory management” OR “managing inventory” OR “inventory control” OR “controlling inventory” OR “stock management” OR “managing stock” OR “stock control” OR “controlling stock” OR “supply management” OR “managing supply”  AND Bangladesh OR Bhutan OR Democratic People’s Republic of Korea OR North Korea OR India OR Indonesia OR Maldives OR Myanmar OR Nepal OR Sri Lanka OR Thailand OR Timor-Leste OR East Timor OR Australia OR Brunei Darussalam OR Cambodia OR China OR Cook Islands OR Fiji OR Japan OR Kiribati OR Lao People's Democratic Republic OR Laos OR Malaysia OR Marshall Islands OR Micronesia OR Mongolia OR Nauru OR New Zealand OR Niue OR Palau OR Papua New Guinea OR Philippines OR Republic of Korea OR South Korea OR Samoa OR Singapore OR Solomon Islands OR Tokelau OR Tonga OR Tuvalu OR Vanuatu OR Vietnam |
| Emerald Insight | hospital OR “healthcare facility”  AND medicine OR medication OR drug OR pharmacy OR pharmaceutical    AND “inventory management” OR “managing inventory” OR “inventory control” OR “controlling inventory” OR “stock management” OR “managing stock” OR “stock control” OR “controlling stock” OR “supply management” OR “managing supply”  AND Bangladesh OR Bhutan OR Democratic People’s Republic of Korea OR North Korea OR India OR Indonesia OR Maldives OR Myanmar OR Nepal OR Sri Lanka OR Thailand OR Timor-Leste OR East Timor OR Australia OR Brunei Darussalam OR Cambodia OR China OR Cook Islands OR Fiji OR Japan OR Kiribati OR Lao People's Democratic Republic OR Laos OR Malaysia OR Marshall Islands OR Micronesia OR Mongolia OR Nauru OR New Zealand OR Niue OR Palau OR Papua New Guinea OR Philippines OR Republic of Korea OR South Korea OR Samoa OR Singapore OR Solomon Islands OR Tokelau OR Tonga OR Tuvalu OR Vanuatu OR Vietnam |
| Google Scholar | hospital OR “healthcare facility”  AND medicine OR drug OR pharmacy AND “inventory management” OR “inventory control” OR “stock management” OR “stock control” OR “supply management”  AND Bangladesh OR Bhutan OR Democratic People’s Republic of Korea OR North Korea OR India OR Indonesia OR Maldives OR Myanmar OR Nepal OR Sri Lanka OR Thailand OR Timor-Leste OR East Timor OR Australia OR Brunei Darussalam OR Cambodia OR China OR Cook Islands OR Fiji OR Japan OR Kiribati OR Lao People's Democratic Republic OR Laos OR Malaysia OR Marshall Islands OR Micronesia OR Mongolia OR Nauru OR New Zealand OR Niue OR Palau OR Papua New Guinea OR Philippines OR Republic of Korea OR South Korea OR Samoa OR Singapore OR Solomon Islands OR Tokelau OR Tonga OR Tuvalu OR Vanuatu OR Vietnam |
| Google Search Engine | hospital OR “healthcare facility”  AND medicine OR drug OR pharmacy AND “inventory management” OR “inventory control” OR “stock management” OR “stock control” OR “supply management”  AND Bangladesh OR Bhutan OR Democratic People’s Republic of Korea OR North Korea OR India OR Indonesia OR Maldives OR Myanmar OR Nepal OR Sri Lanka OR Thailand OR Timor-Leste OR East Timor OR Australia OR Brunei Darussalam OR Cambodia OR China OR Cook Islands OR Fiji OR Japan OR Kiribati OR Lao People's Democratic Republic OR Laos OR Malaysia OR Marshall Islands OR Micronesia OR Mongolia OR Nauru OR New Zealand OR Niue OR Palau OR Papua New Guinea OR Philippines OR Republic of Korea OR South Korea OR Samoa OR Singapore OR Solomon Islands OR Tokelau OR Tonga OR Tuvalu OR Vanuatu OR Vietnam |
