# Supplementary material for: Pharmacy inventory management practices and constraints in Asia-Pacific hospitals: a systematic review and qualitative synthesis
Source: J Pharm Policy Pract. 2026 Jul 28;19(1):2701922. doi: 10.1080/20523211.2026.2701922 (PMC13417656; doi:10.1080/20523211.2026.2701922)
Supplement: Appendix 1 List of countries.docx [file JPPP_A_2701922_SM7074.docx]

# Appendix 1. List of countries and territories in the Asia-Pacific region defined in this review.

This review defines the Asia–Pacific region using the World Health Organization’s classifications for the South-East Asia and Western Pacific regions.

Countries and territories included:

Australia, Bangladesh, Bhutan, Brunei Darussalam, Cambodia, China, Cook Islands, Democratic People’s Republic of Korea, Fiji, India, Indonesia, Japan, Kiribati, Lao People’s Democratic Republic, Malaysia, Maldives, Marshall Islands, Micronesia, Mongolia, Myanmar, Nauru, Nepal, New Zealand, Niue, Palau, Papua New Guinea, the Philippines, Republic of Korea, Samoa, Singapore, Solomon Islands, Sri Lanka, Thailand, Tokelau, Tonga, Timor-Leste, Tuvalu, Vanuatu, and Vietnam.
